# 2,4-Diamino-5-(4-chloro­phen­yl)-6-ethyl­pyrimidin-1-ium 2-propanamido­benzoate

**DOI:** 10.1107/S1600536811035501

**Published:** 2011-09-14

**Authors:** Sampath Natarajan, Rita Mathews

**Affiliations:** a Department of Advanced Technology Fusion, Konkuk University, 1 Hwayang-dong, Gwangjin-gu, Seoul 143 701, Republic of Korea

## Abstract

In the title salt, C_12_H_14_ClN_4_^+^·C_10_H_10_NO_3_^−^, zwitterionic N—H⋯O inter­actions form an *R*_2_^2^(8) ring. The crystal structure is stabilized by N—H⋯O and N—H⋯N hydrogen bonds involving two different eight-membered rings. An N—H⋯O inter­action occurs between the pyrimidine ring (donor) and carboxyl­ate group (acceptor) while the other ring is formed by N—H⋯N inter­actions, which form a dimer between two symmetry-related salts. An intra­molecular N—H⋯O hydrogen bond forms a six-membered ring in the benzoate. Inter­molecular C—H⋯O inter­actions are also observed.

## Related literature

For amino­pyrimidine carboxyl­ates, see: Chinnakali *et al.* (1999 ▶); Lynch & Jones (2004 ▶); Stanley *et al.* (2005 ▶). For amino­pyrimidine and benzoic acid adducts, see: Balasub­ram­ani *et al.* (2005 ▶, 2006 ▶); Thanigaimani *et al.* (2006 ▶, 2007 ▶). For hydrogen bonding in mol­ecular recognition and crystal engin­eering, see: Desiraju (1989 ▶). For puckering and asymmetry parameters, see: Cremer & Pople, (1975 ▶); Nardelli (1995 ▶).
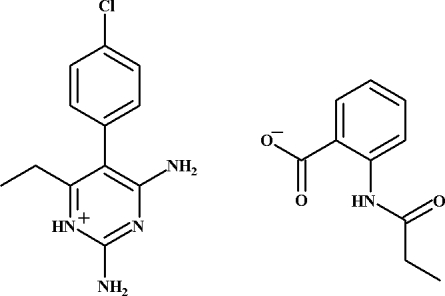


## Experimental

### 

#### Crystal data

C_12_H_14_ClN_4_^+^·C_10_H_10_NO_3_^−^
*M* *_r_* = 441.91
Monoclinic, 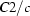

*a* = 22.144 (3) Å
*b* = 9.4915 (14) Å
*c* = 21.844 (3) Å
β = 99.071 (3)°
*V* = 4533.7 (12) Å^3^
*Z* = 8
Mo *K*α radiation
μ = 0.20 mm^−1^
*T* = 293 K
0.50 × 0.45 × 0.42 mm

#### Data collection

Bruker SMART APEX CCD area-detector diffractometer
17933 measured reflections
5077 independent reflections
2719 reflections with *I* > 2σ(*I*)
*R*_int_ = 0.039

#### Refinement

*R*[*F* ^2^ > 2σ(*F* ^2^)] = 0.088
*wR*(*F* ^2^) = 0.228
*S* = 1.06
5077 reflections
281 parameters
H-atom parameters constrained
Δρ_max_ = 0.29 e Å^−3^
Δρ_min_ = −0.24 e Å^−3^

### 

Data collection: *APEX2* (Bruker, 2004 ▶); cell refinement: *SAINT* (Bruker, 2004 ▶); data reduction: *SAINT*; program(s) used to solve structure: *SHELXS97* (Sheldrick, 2008 ▶); program(s) used to refine structure: *SHELXL97* (Sheldrick, 2008 ▶); molecular graphics: *ORTEP-3* (Farrugia, 1997 ▶); software used to prepare material for publication: *PLATON* (Spek, 2009 ▶).

## Supplementary Material

Crystal structure: contains datablock(s) I, global. DOI: 10.1107/S1600536811035501/ff2025sup1.cif

Structure factors: contains datablock(s) I. DOI: 10.1107/S1600536811035501/ff2025Isup2.hkl

Supplementary material file. DOI: 10.1107/S1600536811035501/ff2025Isup3.cml

Additional supplementary materials:  crystallographic information; 3D view; checkCIF report

## Figures and Tables

**Table 1:** Hydrogen-bond geometry (Å, °)

*D*—H⋯*A*	*D*—H	H⋯*A*	*D*⋯*A*	*D*—H⋯*A*
N5—H5⋯O2	0.86	1.89	2.605 (4)	140
N2—H2*A*⋯O3^i^	0.86	2.15	2.977 (4)	163
N4—H4*A*⋯N1^ii^	0.86	2.16	2.988 (4)	163
N3—H3⋯O1^iii^	0.86	1.80	2.660 (4)	178
C14—H14*C*⋯O1^iii^	0.96	2.53	3.323 (6)	140
N2—H2*B*⋯O2^iii^	0.86	1.91	2.746 (4)	164
N4—H4*B*⋯O3^iv^	0.86	2.35	3.017 (3)	134

Symmetry codes: (i) 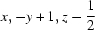; (ii) 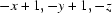; (iii) 
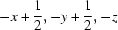
; (iv) 
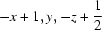
.

**Fig. 1.**
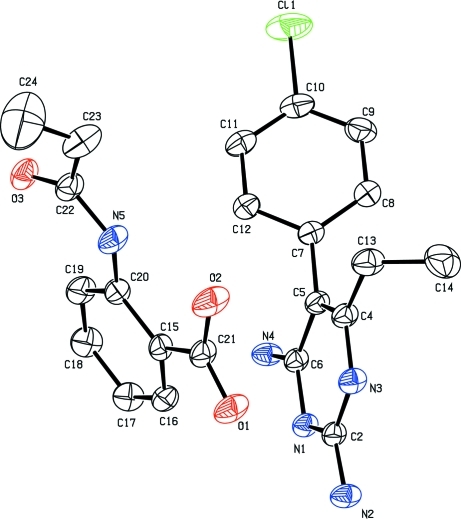
ORTEP diagram of the title molecule with the atom numbering scheme. Displacement ellipsoid are drawn at 30% probability level.

**Fig. 2.**
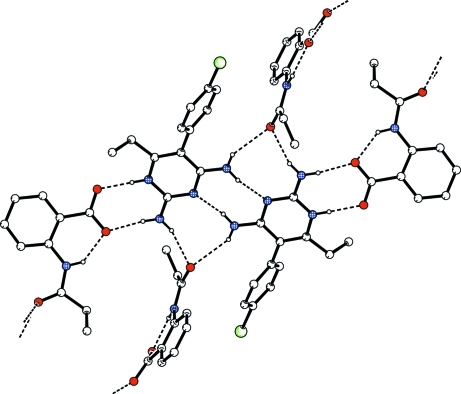
Dimer interaction between the symmetry related salts of title compound. Dashed lines indicate the intra and intermolecular hydrogen bonds.

**Fig. 3.**
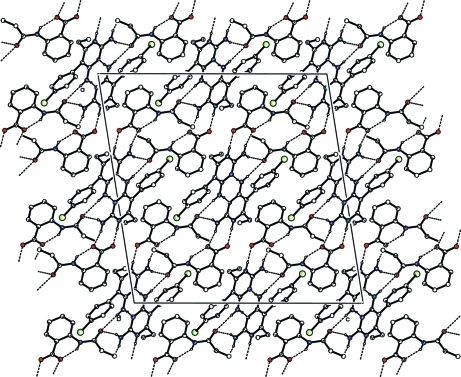
Packing diagram of the title compound viewed down the b-axis. Dashed lines indicate the intra and intermolecular interactions between the molecules.

## supplementary crystallographic information

### Comment

Aminopyrimidine-Carboxylate interactions are important since they are involved in protein-nucleic acids recognition and protein-drug binding. Hydrogen bonding plays a key role in molecular recognition and crystal engineering research (Desiraju, 1989). In general, aminopyrimidines posses self complementary hydrogen-bonded motifs forming a base pair which itself is a unique property. The adducts of carboxylic acid with 2-aminopyrimidine system form a graph-set motif *R*_2_^2^(8) (Lynch & Jones, 2004). This motif is very robust in aminopyrimidine-carboxylic acid/carboxylates systems. The crystal structures of many aminopyrimidine carboxylates (Stanley *et al.*, 2005) and co-crystal structures (Chinnakali *et al.*,1999) have been reported. Many structures of aminopyrimidine and benzoic acid adducts are also have been reported. Few of them are 2-amino-4,6-dimethoxy pyrimidine: 4-aminobenzoic acid (Thanigaimani *et al.*, 2006), 2-amino-4,6-dimethoxypyrimidine: phthalic acid (Thanigaimani *et al.*, 2007), 2-amino-4,6-dimethylpyrimidine: cinnamic acid (Balasubramani *et al.*, 2005) and 2-amino-4,6-dimethylpyrimidine: 4-hydroxybenzoic acid (Balasubramani *et al.*, 2006). All these reported structures have common features of heterosynthone formation. In the present study we report a salt (1:1) namely, 2,4-diamino-5-(4-chlorophenyl)-6-ethylpyrimidin-1-ium 2-propanamidobenzoate which forms a zwitterionic interaction between the molecules of aminopyrimidine and benzoate exhibit a motif *R*_2_^2^(8) ring.

The asymmetric unit of crystal contains a single molecule of each component of salt (Fig. 1). Interactions are found between the salt of aminopyrimidin-1-ium and benzoate *via* hydrogen bonds N2—H2B···O2 and N3—H3···O1 (Fig. 2). Here the pyrimidine acts as a donor which donates two H atoms to carboxylate O atoms (acceptor). In addition, a dimeric interaction through centre of inverted symmetry related salts *via* a hydrogen bond N4—H4A···N1 (Fig. 2) forms an eight membered ring. The dihedral angle between the rings, 4-chlorophenyl and 2,4-diaminopyrimidine is 63.8 (1)°. This value is higher than that in a biphenyl ring system. This may be due to the substitution of ethyl and amine groups at C4 and C6, respectively. An extended moiety of propanamido group is slightly deviating from the plane of benzoate moiety and the dihedral angle between these two is 10.8 (1)° (Cremer & Pople, 1975; Nardelli, 1995).

The packing diagram of the molecule viewed down *b*-axis is shown in Fig. 3. Two symmetry related molecules of salt form the dimer and organize as a sheet. This sheet like dimers are connected through the hydrogen bonds N4—H4B···O3 & N2—H2A···O3 interactions. In addition, a six membered ring is formed by an intra-molecular interaction (N5—H5···O2)in benzoate molecule which also controls the molecules in crystal packing. Molecular packing is stabilized by many N—H···O and N—H···N types intra and intermolecular interactions (Table 1, Fig. 2).

### Experimental

A hot methanolic solution (20 ml) of 2,4-diamino-5-(4-chlorophenyl)-6- ethylpyrimidine and 2-(propanoylamino)benzoic acid in the ratio of 1:1 was warmed for 0.5 h over a water bath. The mixture was cooled slowly and kept at room temperature and after a few days, colourless crystals were obtained

### Refinement

H atoms were positioned geometrically and refined using a riding model with C—H = 0.93 Å for aromatic H, 0.97 Å for methylene, 0.96 Å for methyl H atoms and for aromatic NH_2_ and N—H = 0.86 Å. The *U*_iso_ parameters for H atoms were constrained to be 1.5*U*_eq_ of the carrier atom for the methyl H atoms and 1.2*U*_eq_ of the carrier atom for the remaining H atoms.

### Crystal data

**Table:**

C_12_H_14_ClN_4_^+^·C_10_H_10_NO_3_^−^	*F*(000) = 1856
*M**_r_* = 441.91	*D*_x_ = 1.295 Mg m^−^^3^
Monoclinic, *C*2/*c*	Mo *K*α radiation, λ = 0.71073 Å
*a* = 22.144 (3) Å	Cell parameters from 17933 reflections
*b* = 9.4915 (14) Å	θ = 1.9–28.0°
*c* = 21.844 (3) Å	µ = 0.20 mm^−^^1^
β = 99.071 (3)°	*T* = 293 K
*V* = 4533.7 (12) Å^3^	Block, colorless
*Z* = 8	0.50 × 0.45 × 0.42 mm

### Data collection

**Table:**

Bruker SMART APEX CCD area-detector diffractometer	2719 reflections with *I* > 2σ(*I*)
Radiation source: fine-focus sealed tube	*R*_int_ = 0.039
graphite	θ_max_ = 28.0°, θ_min_ = 1.9°
ω scans	*h* = −28→29
17933 measured reflections	*k* = −10→12
5077 independent reflections	*l* = −28→28

### Refinement

**Table:**

Refinement on *F*^2^	Primary atom site location: structure-invariant direct methods
Least-squares matrix: full	Secondary atom site location: difference Fourier map
*R*[*F*^2^ > 2σ(*F*^2^)] = 0.088	Hydrogen site location: inferred from neighbouring sites
*wR*(*F*^2^) = 0.228	H-atom parameters constrained
*S* = 1.06	*w* = 1/[σ^2^(*F*_o_^2^) + (0.1005*P*)^2^ + 1.940*P*] where *P* = (*F*_o_^2^ + 2*F*_c_^2^)/3
5077 reflections	(Δ/σ)_max_ = 0.002
281 parameters	Δρ_max_ = 0.29 e Å^−^^3^
0 restraints	Δρ_min_ = −0.24 e Å^−^^3^

### Special details

**Table:**

Geometry. All e.s.d.'s (except the e.s.d. in the dihedral angle between two l.s. planes) are estimated using the full covariance matrix. The cell e.s.d.'s are taken into account individually in the estimation of e.s.d.'s in distances, angles and torsion angles; correlations between e.s.d.'s in cell parameters are only used when they are defined by crystal symmetry. An approximate (isotropic) treatment of cell e.s.d.'s is used for estimating e.s.d.'s involving l.s. planes.
Refinement. Refinement of *F*^2^ against ALL reflections. The weighted *R*-factor *wR* and goodness of fit *S* are based on *F*^2^, conventional *R*-factors *R* are based on *F*, with *F* set to zero for negative *F*^2^. The threshold expression of *F*^2^ > σ(*F*^2^) is used only for calculating *R*-factors(gt) *etc*. and is not relevant to the choice of reflections for refinement. *R*-factors based on *F*^2^ are statistically about twice as large as those based on *F*, and *R*- factors based on ALL data will be even larger.

### Fractional atomic coordinates and isotropic or equivalent isotropic displacement parameters (Å^2^)

**Table:**

	*x*	*y*	*z*	*U*_iso_*/*U*_eq_	
Cl1	0.62790 (7)	−0.12826 (18)	0.25493 (7)	0.1465 (7)	
O1	0.25218 (12)	0.3854 (3)	0.05381 (12)	0.0926 (9)	
O2	0.25430 (14)	0.2607 (4)	0.13892 (13)	0.1067 (11)	
O3	0.37717 (11)	0.3960 (3)	0.33389 (11)	0.0809 (7)	
N1	0.43891 (11)	0.3633 (3)	−0.01402 (11)	0.0567 (7)	
N2	0.34901 (13)	0.3749 (3)	−0.08192 (12)	0.0708 (8)	
H2A	0.3626	0.4469	−0.0994	0.085*	
H2B	0.3130	0.3430	−0.0953	0.085*	
N3	0.36043 (12)	0.2008 (3)	−0.00894 (12)	0.0640 (7)	
H3	0.3237	0.1738	−0.0228	0.077*	
N4	0.52835 (12)	0.3481 (3)	0.05255 (12)	0.0654 (7)	
H4A	0.5399	0.4220	0.0348	0.078*	
H4B	0.5525	0.3093	0.0825	0.078*	
N5	0.32811 (13)	0.3417 (3)	0.23743 (12)	0.0763 (9)	
H5	0.2990	0.2900	0.2188	0.092*	
C2	0.38321 (14)	0.3137 (3)	−0.03469 (14)	0.0567 (8)	
C4	0.39373 (15)	0.1283 (4)	0.03825 (14)	0.0620 (9)	
C5	0.45211 (14)	0.1711 (3)	0.06071 (13)	0.0562 (8)	
C6	0.47322 (14)	0.2937 (3)	0.03366 (13)	0.0544 (8)	
C7	0.49398 (15)	0.0933 (4)	0.10941 (14)	0.0590 (8)	
C8	0.51370 (18)	−0.0416 (4)	0.09988 (16)	0.0780 (10)	
H8	0.4995	−0.0873	0.0627	0.094*	
C9	0.5542 (2)	−0.1100 (4)	0.1447 (2)	0.0925 (13)	
H9	0.5666	−0.2014	0.1378	0.111*	
C10	0.57609 (18)	−0.0431 (5)	0.19934 (18)	0.0828 (11)	
C11	0.55717 (17)	0.0894 (5)	0.21009 (16)	0.0770 (11)	
H11	0.5722	0.1341	0.2473	0.092*	
C12	0.51563 (16)	0.1586 (4)	0.16609 (15)	0.0676 (9)	
H12	0.5021	0.2484	0.1742	0.081*	
C13	0.36238 (19)	0.0048 (4)	0.06172 (18)	0.0857 (12)	
H13A	0.3875	−0.0302	0.0990	0.103*	
H13B	0.3238	0.0360	0.0729	0.103*	
C14	0.3503 (3)	−0.1118 (5)	0.0168 (3)	0.1222 (18)	
H14A	0.3300	−0.1870	0.0348	0.183*	
H14B	0.3883	−0.1455	0.0064	0.183*	
H14C	0.3247	−0.0788	−0.0200	0.183*	
C15	0.33329 (14)	0.4293 (4)	0.13458 (15)	0.0596 (8)	
C16	0.36330 (15)	0.5078 (4)	0.09550 (16)	0.0663 (9)	
H16	0.3467	0.5132	0.0537	0.080*	
C17	0.41712 (16)	0.5788 (4)	0.11626 (18)	0.0754 (10)	
H17	0.4364	0.6313	0.0891	0.090*	
C18	0.44141 (17)	0.5700 (4)	0.17795 (19)	0.0833 (11)	
H18	0.4777	0.6169	0.1927	0.100*	
C19	0.41304 (16)	0.4932 (4)	0.21807 (18)	0.0774 (10)	
H19	0.4303	0.4888	0.2597	0.093*	
C20	0.35892 (15)	0.4216 (4)	0.19784 (15)	0.0620 (8)	
C21	0.27569 (16)	0.3551 (4)	0.10749 (16)	0.0687 (9)	
C22	0.33649 (17)	0.3327 (4)	0.29967 (16)	0.0758 (10)	
C23	0.2921 (2)	0.2325 (7)	0.3252 (2)	0.1161 (17)	
H23A	0.2583	0.2126	0.2924	0.139*	
H23B	0.3131	0.1442	0.3363	0.139*	
C24	0.2684 (5)	0.2828 (11)	0.3771 (4)	0.247 (6)	
H24A	0.2412	0.2139	0.3898	0.371*	
H24B	0.2465	0.3689	0.3665	0.371*	
H24C	0.3014	0.2999	0.4104	0.371*	

### Atomic displacement parameters (Å^2^)

**Table:**

	*U*^11^	*U*^22^	*U*^33^	*U*^12^	*U*^13^	*U*^23^
Cl1	0.1356 (12)	0.1739 (14)	0.1179 (10)	0.0501 (10)	−0.0176 (8)	0.0603 (10)
O1	0.0793 (17)	0.122 (2)	0.0646 (15)	−0.0330 (16)	−0.0247 (13)	0.0108 (15)
O2	0.097 (2)	0.132 (3)	0.0758 (17)	−0.0575 (19)	−0.0334 (15)	0.0152 (17)
O3	0.0685 (15)	0.104 (2)	0.0626 (14)	0.0039 (14)	−0.0119 (12)	−0.0153 (14)
N1	0.0503 (14)	0.0625 (16)	0.0522 (14)	−0.0062 (13)	−0.0077 (11)	0.0027 (12)
N2	0.0611 (16)	0.0756 (19)	0.0667 (17)	−0.0151 (15)	−0.0177 (14)	0.0160 (15)
N3	0.0519 (15)	0.0730 (19)	0.0613 (16)	−0.0176 (14)	−0.0093 (12)	0.0036 (14)
N4	0.0542 (15)	0.0687 (18)	0.0675 (17)	−0.0089 (14)	−0.0082 (13)	0.0185 (14)
N5	0.0629 (17)	0.100 (2)	0.0574 (17)	−0.0223 (17)	−0.0183 (14)	−0.0015 (16)
C2	0.0562 (19)	0.0574 (19)	0.0522 (17)	−0.0120 (16)	−0.0045 (15)	−0.0012 (15)
C4	0.062 (2)	0.067 (2)	0.0534 (18)	−0.0103 (17)	−0.0020 (15)	0.0032 (16)
C5	0.0576 (18)	0.061 (2)	0.0484 (16)	−0.0032 (16)	0.0024 (14)	0.0017 (14)
C6	0.0513 (17)	0.060 (2)	0.0494 (17)	−0.0062 (16)	0.0014 (14)	−0.0001 (14)
C7	0.0579 (18)	0.065 (2)	0.0528 (18)	−0.0041 (16)	0.0051 (15)	0.0059 (15)
C8	0.098 (3)	0.072 (3)	0.061 (2)	0.006 (2)	0.005 (2)	0.0003 (18)
C9	0.116 (4)	0.078 (3)	0.085 (3)	0.033 (3)	0.020 (3)	0.019 (2)
C10	0.075 (2)	0.104 (3)	0.068 (2)	0.014 (2)	0.005 (2)	0.026 (2)
C11	0.072 (2)	0.099 (3)	0.055 (2)	−0.005 (2)	−0.0049 (18)	0.009 (2)
C12	0.069 (2)	0.073 (2)	0.058 (2)	0.0008 (18)	0.0028 (17)	0.0016 (17)
C13	0.081 (3)	0.089 (3)	0.080 (2)	−0.021 (2)	−0.009 (2)	0.016 (2)
C14	0.132 (4)	0.085 (3)	0.139 (4)	−0.026 (3)	−0.013 (4)	0.007 (3)
C15	0.0490 (17)	0.063 (2)	0.0607 (19)	0.0016 (16)	−0.0089 (15)	−0.0106 (16)
C16	0.061 (2)	0.076 (2)	0.0568 (18)	−0.0003 (18)	−0.0051 (16)	−0.0004 (17)
C17	0.055 (2)	0.084 (3)	0.084 (3)	−0.0058 (19)	0.0027 (18)	0.006 (2)
C18	0.061 (2)	0.090 (3)	0.091 (3)	−0.017 (2)	−0.013 (2)	0.002 (2)
C19	0.065 (2)	0.086 (3)	0.071 (2)	−0.012 (2)	−0.0202 (18)	0.000 (2)
C20	0.0519 (18)	0.066 (2)	0.0625 (19)	−0.0002 (17)	−0.0091 (15)	−0.0040 (17)
C21	0.062 (2)	0.078 (2)	0.060 (2)	−0.0134 (19)	−0.0081 (17)	−0.0059 (19)
C22	0.065 (2)	0.095 (3)	0.061 (2)	0.008 (2)	−0.0099 (18)	−0.005 (2)
C23	0.100 (3)	0.174 (5)	0.073 (3)	−0.012 (3)	0.009 (2)	0.015 (3)
C24	0.312 (13)	0.252 (10)	0.206 (9)	−0.147 (10)	0.126 (9)	−0.044 (8)

### Geometric parameters (Å, °)

**Table:**

Cl1—C10	1.734 (4)	C10—C11	1.357 (6)
O1—C21	1.240 (4)	C11—C12	1.387 (5)
O2—C21	1.265 (4)	C11—H11	0.9300
O3—C22	1.232 (4)	C12—H12	0.9300
N1—C2	1.331 (4)	C13—C14	1.475 (6)
N1—C6	1.360 (4)	C13—H13A	0.9700
N2—C2	1.315 (4)	C13—H13B	0.9700
N2—H2A	0.8600	C14—H14A	0.9600
N2—H2B	0.8600	C14—H14B	0.9600
N3—C2	1.345 (4)	C14—H14C	0.9600
N3—C4	1.356 (4)	C15—C16	1.380 (5)
N3—H3	0.8600	C15—C20	1.410 (4)
N4—C6	1.330 (4)	C15—C21	1.495 (5)
N4—H4A	0.8600	C16—C17	1.382 (5)
N4—H4B	0.8600	C16—H16	0.9300
N5—C22	1.346 (4)	C17—C18	1.372 (5)
N5—C20	1.405 (4)	C17—H17	0.9300
N5—H5	0.8600	C18—C19	1.366 (5)
C4—C5	1.370 (4)	C18—H18	0.9300
C4—C13	1.494 (5)	C19—C20	1.388 (5)
C5—C6	1.417 (4)	C19—H19	0.9300
C5—C7	1.492 (4)	C22—C23	1.534 (6)
C7—C8	1.379 (5)	C23—C24	1.406 (9)
C7—C12	1.400 (4)	C23—H23A	0.9700
C8—C9	1.381 (5)	C23—H23B	0.9700
C8—H8	0.9300	C24—H24A	0.9600
C9—C10	1.372 (6)	C24—H24B	0.9600
C9—H9	0.9300	C24—H24C	0.9600
			
C2—N1—C6	117.6 (3)	C14—C13—H13B	108.8
C2—N2—H2A	120.0	C4—C13—H13B	108.8
C2—N2—H2B	120.0	H13A—C13—H13B	107.7
H2A—N2—H2B	120.0	C13—C14—H14A	109.5
C2—N3—C4	121.8 (3)	C13—C14—H14B	109.5
C2—N3—H3	119.1	H14A—C14—H14B	109.5
C4—N3—H3	119.1	C13—C14—H14C	109.5
C6—N4—H4A	120.0	H14A—C14—H14C	109.5
C6—N4—H4B	120.0	H14B—C14—H14C	109.5
H4A—N4—H4B	120.0	C16—C15—C20	118.4 (3)
C22—N5—C20	130.8 (3)	C16—C15—C21	118.3 (3)
C22—N5—H5	114.6	C20—C15—C21	123.4 (3)
C20—N5—H5	114.6	C15—C16—C17	122.3 (3)
N2—C2—N1	119.9 (3)	C15—C16—H16	118.8
N2—C2—N3	118.2 (3)	C17—C16—H16	118.8
N1—C2—N3	121.9 (3)	C18—C17—C16	118.5 (4)
N3—C4—C5	119.4 (3)	C18—C17—H17	120.8
N3—C4—C13	115.6 (3)	C16—C17—H17	120.8
C5—C4—C13	125.0 (3)	C19—C18—C17	120.9 (3)
C4—C5—C6	116.6 (3)	C19—C18—H18	119.5
C4—C5—C7	123.6 (3)	C17—C18—H18	119.5
C6—C5—C7	119.7 (3)	C18—C19—C20	121.2 (3)
N4—C6—N1	115.1 (3)	C18—C19—H19	119.4
N4—C6—C5	122.4 (3)	C20—C19—H19	119.4
N1—C6—C5	122.5 (3)	C19—C20—N5	123.1 (3)
C8—C7—C12	118.2 (3)	C19—C20—C15	118.7 (3)
C8—C7—C5	121.9 (3)	N5—C20—C15	118.1 (3)
C12—C7—C5	119.9 (3)	O1—C21—O2	122.7 (3)
C7—C8—C9	121.0 (4)	O1—C21—C15	118.0 (3)
C7—C8—H8	119.5	O2—C21—C15	119.2 (3)
C9—C8—H8	119.5	O3—C22—N5	123.6 (4)
C10—C9—C8	120.0 (4)	O3—C22—C23	122.0 (3)
C10—C9—H9	120.0	N5—C22—C23	114.4 (3)
C8—C9—H9	120.0	C24—C23—C22	115.1 (6)
C11—C10—C9	120.2 (4)	C24—C23—H23A	108.5
C11—C10—Cl1	120.0 (3)	C22—C23—H23A	108.5
C9—C10—Cl1	119.9 (4)	C24—C23—H23B	108.5
C10—C11—C12	120.5 (4)	C22—C23—H23B	108.5
C10—C11—H11	119.7	H23A—C23—H23B	107.5
C12—C11—H11	119.7	C23—C24—H24A	109.5
C11—C12—C7	120.0 (4)	C23—C24—H24B	109.5
C11—C12—H12	120.0	H24A—C24—H24B	109.5
C7—C12—H12	120.0	C23—C24—H24C	109.5
C14—C13—C4	114.0 (4)	H24A—C24—H24C	109.5
C14—C13—H13A	108.8	H24B—C24—H24C	109.5
C4—C13—H13A	108.8		
			
C6—N1—C2—N2	−178.0 (3)	C10—C11—C12—C7	−1.6 (5)
C6—N1—C2—N3	1.5 (5)	C8—C7—C12—C11	1.9 (5)
C4—N3—C2—N2	177.3 (3)	C5—C7—C12—C11	−176.4 (3)
C4—N3—C2—N1	−2.2 (5)	N3—C4—C13—C14	67.1 (5)
C2—N3—C4—C5	0.3 (5)	C5—C4—C13—C14	−112.8 (4)
C2—N3—C4—C13	−179.6 (3)	C20—C15—C16—C17	−0.1 (5)
N3—C4—C5—C6	2.0 (5)	C21—C15—C16—C17	−179.3 (3)
C13—C4—C5—C6	−178.1 (3)	C15—C16—C17—C18	0.2 (6)
N3—C4—C5—C7	−175.7 (3)	C16—C17—C18—C19	−0.2 (6)
C13—C4—C5—C7	4.2 (5)	C17—C18—C19—C20	0.1 (6)
C2—N1—C6—N4	−179.8 (3)	C18—C19—C20—N5	−179.8 (3)
C2—N1—C6—C5	1.0 (4)	C18—C19—C20—C15	0.0 (6)
C4—C5—C6—N4	178.1 (3)	C22—N5—C20—C19	10.9 (6)
C7—C5—C6—N4	−4.1 (5)	C22—N5—C20—C15	−168.9 (4)
C4—C5—C6—N1	−2.7 (5)	C16—C15—C20—C19	0.0 (5)
C7—C5—C6—N1	175.0 (3)	C21—C15—C20—C19	179.2 (3)
C4—C5—C7—C8	63.2 (5)	C16—C15—C20—N5	179.9 (3)
C6—C5—C7—C8	−114.4 (4)	C21—C15—C20—N5	−1.0 (5)
C4—C5—C7—C12	−118.5 (4)	C16—C15—C21—O1	−11.5 (5)
C6—C5—C7—C12	63.9 (4)	C20—C15—C21—O1	169.3 (3)
C12—C7—C8—C9	−0.7 (5)	C16—C15—C21—O2	165.7 (3)
C5—C7—C8—C9	177.6 (3)	C20—C15—C21—O2	−13.5 (5)
C7—C8—C9—C10	−0.9 (6)	C20—N5—C22—O3	−2.3 (6)
C8—C9—C10—C11	1.3 (6)	C20—N5—C22—C23	179.6 (4)
C8—C9—C10—Cl1	−178.9 (3)	O3—C22—C23—C24	44.3 (8)
C9—C10—C11—C12	−0.1 (6)	N5—C22—C23—C24	−137.5 (7)
Cl1—C10—C11—C12	−179.8 (3)		

### Hydrogen-bond geometry (Å, °)

**Table:**

*D*—H···*A*	*D*—H	H···*A*	*D*···*A*	*D*—H···*A*
N5—H5···O2	0.86	1.89	2.605 (4)	140
N2—H2A···O3^i^	0.86	2.15	2.977 (4)	163
N4—H4A···N1^ii^	0.86	2.16	2.988 (4)	163
N3—H3···O1^iii^	0.86	1.80	2.660 (4)	178
C14—H14C···O1^iii^	0.96	2.53	3.323 (6)	140
N2—H2B···O2^iii^	0.86	1.91	2.746 (4)	164
N4—H4B···O3^iv^	0.86	2.35	3.017 (3)	134

Symmetry codes: (i) *x*, −*y*+1, *z*−1/2; (ii) −*x*+1, −*y*+1, −*z*; (iii) −*x*+1/2, −*y*+1/2, −*z*; (iv) −*x*+1, *y*, −*z*+1/2.
